# Comparison of Different Varieties on Quality Characteristics and Microbial Activity of Fresh-Cut Pineapple during Storage

**DOI:** 10.3390/foods11182788

**Published:** 2022-09-09

**Authors:** Yage Xing, Xingmei Liao, Haijun Wu, Jiamin Qiu, Rufeng Wan, Xiaomin Wang, Rumeng Yi, Qinglian Xu, Xiaocui Liu

**Affiliations:** 1 Key Laboratory of Grain and Oil Processing and Food Safety of Sichuan Province, College of Food and Bioengineering, Xihua University, Chengdu 610039, China; 2 Key Laboratory of Food Non Thermal Processing, Engineering Technology Research Center of Food Non Thermal Processing, Yibin Xihua University Research Institute, Yibin 644004, China

**Keywords:** fresh cut, pineapples, varieties, shelf life, quality

## Abstract

This study compared the quality and storage characteristics of four pineapple varieties to select those displaying adequate storage resistance and those suitable for freshly cut processing. Four varieties of pineapple, namely *Tainong No.16*, *Tainong No.17*, *Tainong No.11*, and *Bali*, were used to analyze the quality differences in freshly cut pineapple during storage by measuring the quality, physiological indicators, and total microbial count. The results indicated that the nutritional quality and storability of freshly cut pineapples differed significantly among the varieties. During refrigeration at 4 °C, *Tainong No.11* and *Bali* displayed the shortest storage period of 4 d, while *Tainong No.17* and *Tainong No.16* presented storage periods of 5 d and 6 d, respectively. A sensory evaluation indicated that the *Tainong No.16* variety was superior in terms of consumer preference, while the *Bali* slices were generally rated lower than the other cultivars. Additionally, the sensory properties, weight loss, firmness, and ascorbic acid (AA) content of *Tainong No.16* changed the least during storage, with values of 60.75%, 6.48%, 75.15%, and 20.44%, respectively. Overall, the quality order of the four varieties of freshly cut pineapples during storage was: *Tainong No.16* > *Tainong No.17* > *Tainong No.11* > *Bali*. Moreover, two-way ANOVA showed that the main effect of variety and storage time on the storage quality of fresh-cut pineapple was significant (*p* < 0.05). The interaction effect of variety and storage time on other quality characteristics of fresh-cut pineapple was significant (*p* < 0.05) except for Titratable acid (TA) and AA. In conclusion, *Tainong No.16* displayed higher storage potential than the other varieties. The results of this work provide application possibilities to promote the successful processing of pineapple cultivars as freshly cut produce.

## 1. Introduction

*Ananas comosus* (L.) *Merr*, commonly known as pineapple, is a perennial monocotyledonous herb belonging to the bromeliaceae family [1]. Native to South America, it is a tropical fruit with encouraging market potential in the global market. As an agricultural product with high economic value, pineapples are cultivated in China and in various parts of the world such as Thailand, India, Brazil, Mexico, the Philippines, and Malaysia [2]. With more than 100 cultivars, pineapple production ranks third globally, second only to citrus and bananas [3]. In 2017, global pineapple production was approximately 27.4 million tons [4]. By 2028, global pineapple production is expected to grow at an annual rate of 1.9% to 31 million tons [5]. In 2019, the total production of pineapples in China was about 1.733 million tons and represented a crucial economic crop [6].

Pineapples present golden flesh, rich flavor, crispness, and juiciness as fresh products. Nearly 80% of pineapples are consumed fresh in the domestic market, while the rest are processed into canned, juice, and preserved products. Pineapples are nutritionally rich and sweet, with a unique flavor and attractive color, and are available throughout the year. These fruits are popular with consumers since they are also beneficial for alleviating summer heat and quenching thirst while aiding digestion, diarrhea, beauty, slimming, diuresis, and swelling [7,8]. However, the thick skin, large crown, and storage space required by pineapples leads to consumption inconvenience and higher transportation costs. In recent years, the domestic and international markets for freshly cut fruits and vegetables have expanded dramatically due to increasing consumer demand for fresh, convenient, additive-free, minimally processed, and nutritionally safe products [9].

Freshly cut fruits and vegetables, also known as semi-processed, lightly processed, or least processed produce, refer to grading, cleaning, trimming, peeling, cutting, freshness, packaging, and other processes that maintain the fresh state of products for immediate consumption by consumers or the catering industry [10,11]. Improved modern living standards and increased health concerns have rendered freshly cut fruits and vegetables highly popular with consumers, prompting the development of related products and effective processing methods. A wide variety of freshly cut products have been used in retail and foodservice distribution [12]. In China, the commercialization of freshly cut fruits and vegetables started relatively late, with potatoes, apples, broccoli, and cabbage the first to emerge, which were mainly sold to chain restaurants, large supermarkets, and large canteens. Freshly cut pineapple has become one of the most popular products domestically and abroad, showing significant development prospects.

The quality of freshly cut fruits and vegetables can be affected by factors such as variety, cutting techniques, climatic conditions, packaging materials, ripeness, cultivation conditions and storage, of which the pineapple variety represents the primary influencing factor [13,14]. Different fruit and vegetable varieties present differences regarding the most suitable physiological stages for freshly cut processing, directly affecting the sensory properties, storage resistance, nutritional quality, and processing characteristics of the product [15]. Pineapples are also available in different varieties worldwide and are divided into four main categories: *Smooth Cayenne*, *Queen*, *Spanish*, and *hybrids*, based on their morphology, leaf spines, and fruit characteristics [16]. In China, pineapples are widely cultivated in Guangdong, Hainan, Guangxi, Fujian, Yunnan, and Taiwan. Currently, most of the pineapple varieties grown in China have been introduced from abroad, such as *Bali*, *Shenwan*, *thornless cocaine*, and *golden pineapples*. China displays a single pineapple variety structure, and it is estimated that the *Bali* variety accounts for 75% of the pineapple cultivation area. This presents significant challenges regarding production and market consumption, such as high concentration during the production period, which reduces market competitiveness [17]. The current research involving freshly cut pineapple mainly focuses on developing and applying cutting techniques, sterilization technology, packaging methods, aroma components, and preservation technology [18]. Minimal studies are available regarding the evaluation of raw varieties suitable for freshly cut processing. Therefore, the selection of high-quality pineapple varieties with suitable processing characteristics is essential for promoting the economic development of the cultivation areas and adjusting the structure of the pineapple industry [19].

This study used four types of pineapples with a high market share in China as experimental materials to compare the freshly cut storage characteristics of different varieties by analyzing the changes in physicochemical indexes such as sensory properties, color, weight loss, hardness, TA, total soluble solids (TSS), AA, malondialdehyde (MDA), polyphenol oxidase (PPO), polyphenol peroxidase (POD), and the total number of bacterial colonies during storage and to identify the varieties suitable for freshly cut processing. This study promotes the raw material selection of high-quality varieties for freshly cut pineapple processing.

## 2. Materials and Methods

### 2.1. Materials

Equally mature, moderately sized pineapples free from pests and mechanical damage were selected for testing. Four different varieties available in the Chinese market, namely *Tainong No.16*, *Tainong No.17*, *Tainong No.11*, and *Bali*, were purchased from local pineapple growers and transported to the post-harvest laboratory of the University of Xihua. The metaphosphoric acid, oxalic acid, sodium bicarbonate, AA standard, sodium hydroxide, phenolphthalein, glacial acetic acid, anhydrous sodium acetate, polyethylene glycol, polyvinylpyrrolidone, Triton X-100, catechol, guaiacol, and H_2_O_2_ were all analytically pure. All reagents were purchased from the Chengdu Kolon Chemical Reagent Co., Ltd. (Chengdu, China).

### 2.2. Minimal Processing

Commercially mature pineapples were selected, and the crown buds, skin, and eyes were removed with a clean pineapple knife. Then, the pineapples were divided into four equal portions and cut into 3–4 mm thick fan slices. They were then placed in plastic fruit boxes with eight slices (about 200 g) per plate and transferred to a refrigerator for storage at 4 °C. Food-grade polyethylene gloves were always used to prevent contamination during the pineapple preparation process. The pineapple slices were sampled at 1 d intervals for the quality analyses, while the day the pineapples were freshly cut was recorded as day 0.

### 2.3. Measurement of the Weight Loss, L* Value and Firmness

During storage of the four pineapple varieties, their weight loss was measured on each sampling day using a JA2003 digital balance (Shanghai, China) with a minimum measurement of 0.01 g. The weight loss of the pineapples before and after storage was recorded separately to assess the weight loss during refrigeration. The results were expressed as the percentage of weight loss, which was calculated using the following equation:

(1) $$Weightloss\;rate\;(\%)=\frac{m_{0}{-m}_{1}}{m_{0}}\times 100$$

where *m*_0_ is the initial weight before storage and *m*_1_ denotes the weight after storage.

The color of the freshly cut pineapples was measured using a Verivide DigiEye. Before each determination of the brightness value, the equipment was standardized by calibrating a standard black and white plate. Five different positions of each pineapple slice were selected for measurement to obtain uniform color measurements, and the L^*^ values were recorded.

The firmness was determined using a TA-XT PLUS texture analyzer according to a method described by Zou et al., with slight modifications [20]. The pineapple slices were placed directly below the cylindrical probe (5 mm diameter, P/5) for puncture testing. The pre-, mid-, and post-measurement velocities were set to 5 mm/s, 2 mm/s, and 2 mm/s, respectively, while the trigger force was set to 5 g. The five points on the surfaces of the freshly cut pineapple were selected randomly, and the flesh hardness was calculated as the average force (N).

### 2.4. Determination of the TSS, TA, and AA Acid Content

The TSS content was measured using a digital refractometer (J1-3A, Shanghai Scientific Instruments, Shanghai, China), which was standardized and calibrated with distilled water. The fruit tissue (10 g) was homogenized and centrifuged at 10,000× *g* for 20 min. The supernatant was collected, after which a drop of juice was placed on the refractometer to obtain the total soluble solid percentage.

The TA was determined via titration, based on a method described by Liu et al., with slight modifications [21]. Here, 1.0 g of the ground pineapple tissue of each species was diluted to 100 mL with distilled water, which was stirred, homogenized, and filtered. The filtrate was transferred to a triangular flask via aspiration, after which two drops of 1% phenolphthalein indicator were added and titrated with the calibrated sodium hydroxide solution. The solution was titrated until it turned pink and did not fade within 0.5 min as the endpoint (pH = 8.1~8.3), after which the amount of sodium hydroxide titrant was recorded. The titration was performed with distilled water instead of filtrate as a blank control. The results were calculated as a coefficient of citric acid, while the TA was expressed as grams of acid per 100 g.

The AA content was analyzed via dichlorophenol-indophenol titration, according to a method described by Tao et al. [22]. A homogenized sample of 10 g of freshly cut pineapple and a metaphosphoric acid solution was weighed into a beaker and diluted in a 100 mL volumetric flask. Then, the subsequent mixture was decolorized with white clay, shaken well, and filtered. Next, 10 mL of filtrate was aspirated into a 50 mL conical flask and titrated with a calibrated 2,6-dichloroindophenol solution until it remained pink for 15 s without fading. The results were expressed as mg/100 g of the sample.

### 2.5. MDA Content

MDA content was measured according to the modified approach delineated by Fan et al. [23]. Here, 1.0 g of freshly cut pineapple and 5.0 mL of 100 g/L TCA solution were ground and homogenized, followed by centrifugation at 4 °C and 10,000 r/min for 20 min, after which the supernatant was collected and stored at a low temperature. Then, 2.0 mL of the supernatant (a blank control tube was filled with 100 g/L TCA solution at 2.0 mL instead of an extract) was mixed with 2.0 mL 0.67% TBA. The mixture was boiled in a water bath for 20 min, removed, cooled in an ice bath, and centrifuged again. The absorbance values of the supernatants were determined at 450 nm, 532 nm, and 600 nm using a spectrophotometer (UV/VIS756-PC, T6, PG General, Beijing, China). The MDA was quantified as follows:

(2) $$MDA\;content\;(\mathrm{nmol}/g)=\frac{[6.452\times ({OD}_{532}-{OD}_{600})-0.559\times {OD}_{450}]\times V_{t}}{M\times V_{s}}$$

where *V_t_* is the volume of the extract solution (mL), *V_s_* denotes the volume of the extract solution contained in the reaction mixture (mL), and *M* represents the mass of the fresh sample (g).

### 2.6. PPO and POD Activity

Acquisition of the enzyme extract: 5 g of fresh-cut pineapple and 5.0 mL of extraction buffer (containing 1 mmol PEG, 4% PVPP, 1% Triton X-100) were mixed, ground into a homogenate in an ice bath, and centrifuged at 4 °C and 10,000 r/min for 30 min. The supernatant was considered the enzyme extract and was stored at 4 °C for measuring the PPO and POD activity.

The PPO activity was evaluated spectrophotometrically using a method delineated by Li et al., with minor modifications [24]. The enzyme activity was determined by measuring the absorbance increase at 420 nm for catechol at 25 °C using a UV_240_ UV/VIS spectrophotometer (Shanghai Sunyu Hengping Scientific Instruments Co., Ltd., Shanghai, China). Then, 4.0 mL of 50 mmol/L acetic acid-sodium acetate buffer at pH 5.5 and 1.0 mL of 50 mmol/L catechol solution were mixed in a test tube, after which 100 uL of the enzyme extract was added. The enzyme activity was expressed as OD_420_/(min·g)^−1^ (fresh weight), while the absorbance change was measured every 30 s at 420 nm at room temperature for 3 min. The 0.001 per min increase in the OD_420_ absorbance value was considered one unit of enzyme activity.

The POD activity was determined via the guaiacol oxidation method according to a method described by Wang et al., with slight modifications [25]. Here, 3.0 mL of 25 mmol/L guaiacol solution and 0.5 mL of the enzyme extract were mixed in a test tube, after which 200 uL of 0.5 mol/L H_2_O_2_ solution was added to obtain the solution. The mixture was shaken well and was poured into a cuvette. The absorbance values were measured using a UV_240_ UV/VIS spectrophotometer (Shanghai Sunyu Hengping Scientific Instruments Co., Ltd., Shanghai, China) at 470 nm every 30 s for 3 min, with distilled water as a blank control. The 0.001 per min increase in the OD_470_ absorbance value was considered one unit of enzyme activity.

### 2.7. Microbiological Analysis

The total microbial count of the freshly cut pineapples was evaluated according to a method described by Yousuf and Srivastava with slight modifications [26]. The freshly cut pineapple (25 g) was homogenized with sterile saline (225 mL) in a blender for 2 min. The homogenized samples were diluted in the order of 1:10, and 1 mL of the sample solution was aspirated for surface coating on the agar plates. The total microbial count was determined via plate count agar after incubation at 37 °C for 24 h. The microbial counts were expressed as the logarithm of colony-forming units per gram (lg CFU/g).

### 2.8. Sensory Evaluation

The sensory evaluation was performed using a method described by Tabassum and Khan with slight modifications [27]. The sensory characteristics of the freshly cut pineapple during storage were determined. Fifteen individuals between the ages of 18 and 50 with experience in sensory evaluation who enjoy and regularly eat pineapple were recruited from the students and staff at the School of Food and Biological Engineering, Xihua University, China. There was equal gender representation in the assessors. The sensory evaluations were conducted in a sensory laboratory equipped with individual sensory booths. The evaluators assessed the samples in terms of taste, smell, color, and texture according to a scale of sensory evaluation criteria on a percentage basis. Each sample was randomly numbered and distributed, and the evaluators were asked to rinse their palates with water between samples. The assessors recorded their responses on a paper scorecard. The specific sensory evaluation criteria are shown in Table 1.

### 2.9. Statistical Analysis

The tests in this study were performed in triplicate. The results were analyzed using SPSS 20.0 software (IBM Corporation, New York, USA) and are expressed as mean ± standard deviation. A one-way analysis of variance (ANOVA), followed by a least significant difference (LSD) test, was used to determine the significant differences (*p* < 0.05) between the treatments. A two-way ANOVA was used to analyze the interaction between storage time and variety on the storage quality of fresh-cut pineapple.

## 3. Results and Discussion

### 3.1. Weight Loss, L* Value, b Value, and Firmness

**Figure 1:**
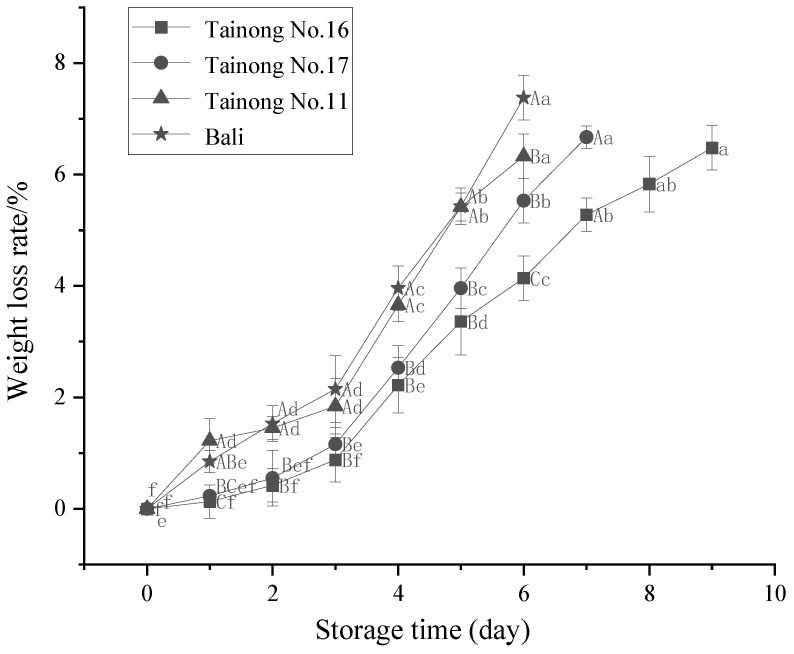
Changes in weight loss rate in freshly cut pineapple during storage. Different capital letters (A–C) indicate significant (*p* < 0.05) differences in weight loss rates between varieties at the same storage time; different lowercase letters (a–f) indicate significant (*p* < 0.05) differences in weight loss rates between the same varieties at different storage times.

Weight loss is an important indicator of the freshness of freshly cut fruits and vegetables [28]. As shown in Figure 1, the weight loss rate of all the freshly cut pineapples increased throughout the storage period. The fruit variety and storage time significantly (*p* < 0.05) affected the weight loss of freshly cut pineapple. During the initial storage period (day 3), *Tainong No.11* and *Bali* displayed faster weight loss rates of 1.84% and 2.15%, respectively. At 6 d of storage, the weight loss rate of *Bali* (7.38%) was significantly (*p* < 0.05) higher than *Tainong No.11* (6.33%), *Tainong No.17* (5.53%), and *Tainong No.16* (4.14%). Throughout the storage period, *Bali* exhibited the fastest increase and most significant weight loss of up to 7.38%, followed by *Tainong No.11*, *Tainong No.17*, and *Tainong No.16*, with weight loss percentages of 6.33%, 6.67%, and 6.48%, respectively. Overall, *Tainong No.16* was more successful in maintaining moisture content during storage than the other varieties. The main and interaction effects of variety and storage time on the quality attributes of fresh-cut pineapple are shown in Table 2. The results showed that the two-way interaction of variety and storage time had a significant (*p* < 0.05) effect on weight loss in fresh-cut pineapple. Partial eta square values indicated the magnitude of the main effect or reciprocal effect. Among the significant effects and interactions, storage time had the largest main effect on weight loss in fresh-cut pineapple.

The variation in the weight loss rates of the different freshly cut pineapple cultivars depended on their physiology, biochemistry, and morphology [29]. These differences were inherent in the raw materials and were related to genetic differences between varieties. This was consistent with the results of previous studies. Sharma and Rao [30] found that the freshly cut products of four different mango varieties displayed various weight loss increase rates, which was related to the storability of the fruit. Similar results were obtained by Oltenacu et al., who reported that the four varieties exhibited different behavior regarding the losses registered during storage with the *Goldspur* variety showing the most significant water loss [31]. This could also be attributed to the mechanical damage suffered after the freshly cut treatment, promoting its respiration, inducing ethylene production, causing significant water loss, and accelerating weight loss [32].

**Figure 2:**
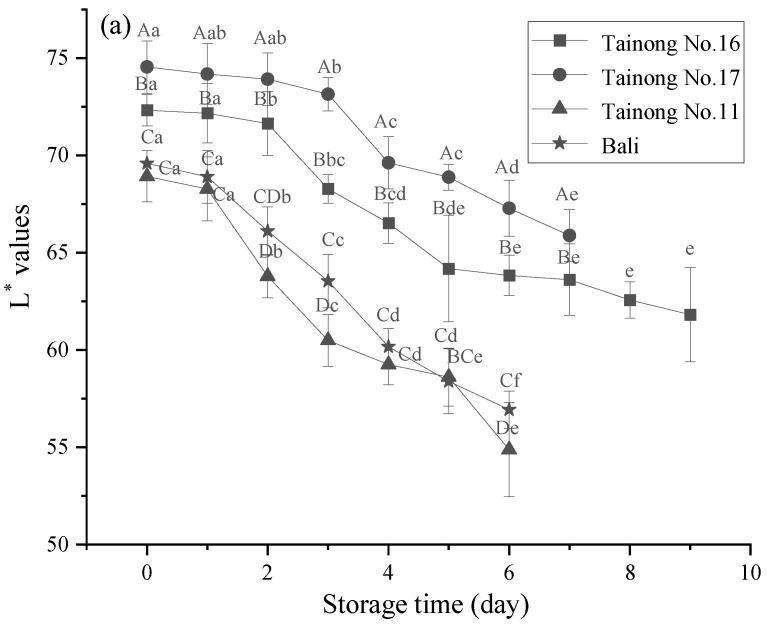

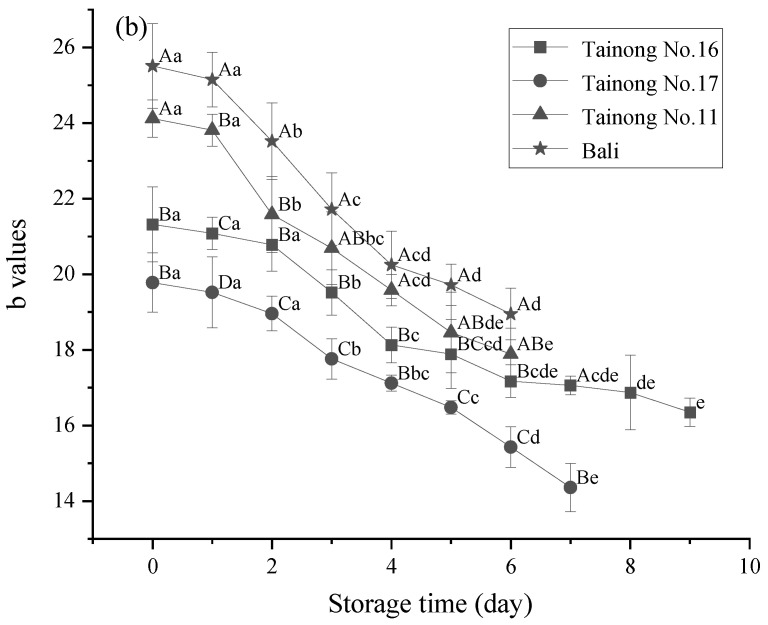
Changes in L* values (**a**) and b values (**b**) in freshly cut pineapple during storage. Different capital letters (A–D) indicate significant (*p* < 0.05) differences in L* values and b values between varieties at the same storage time; different lowercase letters (a–f) indicate significant (*p* < 0.05) differences in L* values and b values between the same varieties at different storage times.

The changes in the L* values and b values of the different freshly cut pineapple varieties are shown in Figure 2. The L* values decreased, confirming that the freshly cut pineapple samples of each type became progressively browner during storage. The initial L* values of each variety differed, with *Tainong No.16* and *Tainong No.17* displaying higher L* values than *Tainong No.11* and *Bali*, corresponding with the pale yellow of the first two varieties and the golden yellow of the remaining two samples. Additionally, the L* value of the latter two varieties decreased rapidly, while that of the first two gradually reduced after 1 d of storage. At the end of the storage period, the L* values of *Tainong No.17* were higher than those of *Tainong No.16*, *Tainong No.11*, and *Bali*, respectively (*p* < 0.05). During storage, the L* values of all four varieties were significantly lower than the initial values (*p* < 0.05). *Tainong No.11* exhibited the most significant decrease in the L* value during storage at 20.31%, while *Tainong No.17* showed the smallest at 11.61%. *Tainong No.17* and *Tainong No.16* showed higher L* values during storage (*p* < 0.05) than *Tainong No.11* and *Bali*. Therefore, *Tainong No.16* and *Tainong No.17* were more resistant to browning and could better maintain color during storage. A positive b value represents yellow, a negative b value represents blue, and a decreasing b value indicates that the yellow color is fading. Significant (*p* < 0.05) differences in L* and b values were found in all varieties of fresh-cut pineapples over time. The color parameter L* and b values of the four varieties significantly (*p* < 0.05) decreased over time, which was directly attributed to the translucency in the fresh-cut pineapple flesh. Similarly, the initial b-values of *Bali* and *Tainong No.11* were significantly (*p* < 0.05) higher than those of *Tainong No.16* and *Tainong No.17*, which also indicated that the former two were golden yellow and the latter two were pale yellow. Furthermore, the results (Table 2) showed that the two-way interaction of variety and storage time had a highly significant effect (*p* ≤ 0.001) on the L* value and a significant effect (*p* < 0.05) on the b value of fresh-cut pineapple, and the main effect of variety on the L* and b values of fresh-cut pineapple was the largest.

Color is used to evaluate the visual quality attributes of fruits and vegetables during the post-harvest storage and distribution process. It can be affected by the biochemical modification of pigment compounds, which is caused by phenolic oxidation, catalyzed by PPO enzymes to form colored melanin [33]. The ultimate browning reaction occurs when the phenolic substrates released after cell membrane damage come into contact with intracellular enzymes, PPO, and POD [34]. Therefore, diverse L* values were evident due to variation in the PPO and POD activity in the different varieties. Different scholars hold different opinions regarding color parameter variation, and the variation pattern in conjunction with storage time remains inconclusive. According to Montero-Calderón et al., the variability of the L* values was lower in the freshly cut pineapple samples of the Gold cultivar [35]. However, Marrero and Kader et al., revealed a decrease in the L* values of the *Smooth Cayenne* variety after storage for 15 d at 5 °C [36]. Moreover, Yang et al., analyzed the correlation between the color changes and carotenoid components in the flesh of the *Bali* and Smooth Cayenne pineapples, indicating that the β-carotene content levels in the different varieties played a substantial role in the flesh color differences of the pineapple fruit [37].

**Figure 3:**
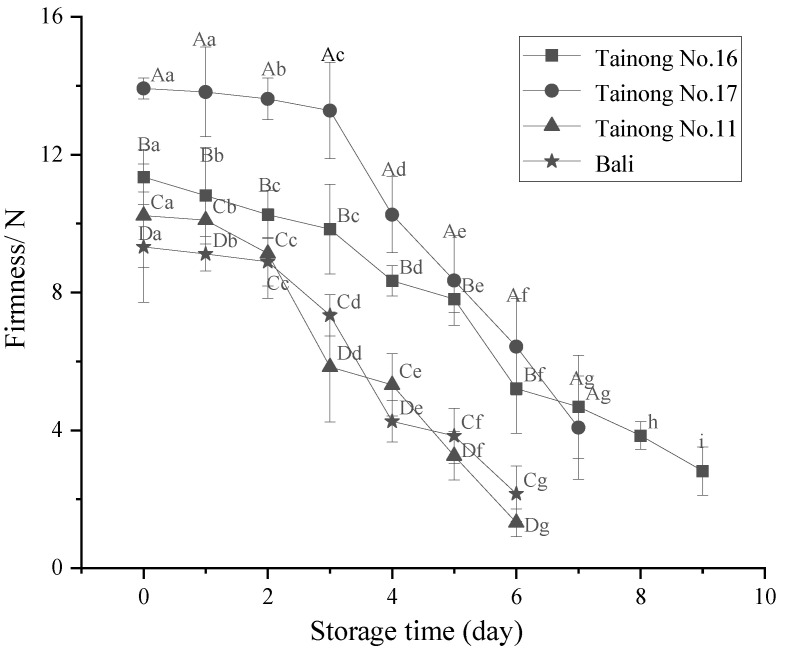
Changes in firmness in freshly cut pineapple during storage. Different capital letters (A–D) indicate significant (*p* < 0.05) differences in firmness between varieties at the same storage time; different lowercase letters (a–i) indicate significant (*p* < 0.05) differences in firmness between the same varieties at different storage times.

The firmness of the different freshly cut pineapple varieties decreased during storage. As shown in Figure 3, the initial firmness of all four freshly cut pineapple varieties differed, with *Tainong No.17* displaying the highest initial firmness, which significantly exceeded the other varieties. From 2 d onwards, the firmness of the four types decreased substantially, demonstrating significant differences *(p* < 0.01) between the samples. At the end of storage, the decreasing order of hardness declined in descending order: *Tainong No.11* > *Bali* > *Tainong No.16* > *Tainong No.17*. In addition, the firmness decline rate of *Tainong No.16* was only 3.60% higher than *Tainong No.17*, indicating that both remained relatively firm during storage, significantly exceeding *Bali* and *Tainong No.11* (*p* < 0.05). The results showed (Table 2) that the two-way interaction of variety and storage time had a highly significant effect (*p* ≤ 0.001) on the hardness of the fresh-cut pineapple, and it had the greatest effect on the hardness of the fresh-cut pineapple.

Firmness can directly reflect the quality and storage resistance of freshly cut pineapple, representing vital indicators for evaluating the maturity and aging of the fruit [38]. An appropriate texture provides freshly cut pineapple with an excellent taste and allows it to withstand adversity to maintain its quality. The texture of fruit or vegetable tissue is affected by various factors, including varietal, environmental, post-harvest handling, and storage elements [39]. The firmness of the freshly cut pineapple samples in this study decreased with storage time. Saftner et al., evaluated the quality of freshly cut *Fuji*, *Granny Smith*, *Pink Lady*, and *GoldRush* apple slices, revealing that different cultivars varied considerably in their rate of textural deterioration [40]. Lin et al., indicated that the firmness of a freshly cut Hami melon decreased with storage time [41]. This phenomenon might be related to reduced water content and metabolic changes. In addition, the rapid growth of microorganisms can also lead to textural loss by converting starch into soluble sugars and pectin into pectic acid, destroying the spatial structure of freshly cut pineapple cells. A previous study showed that fruit softening occurred due to the degradation of cell wall components [42].

### 3.2. TSS, TA, and AA Content

**Figure 4:**
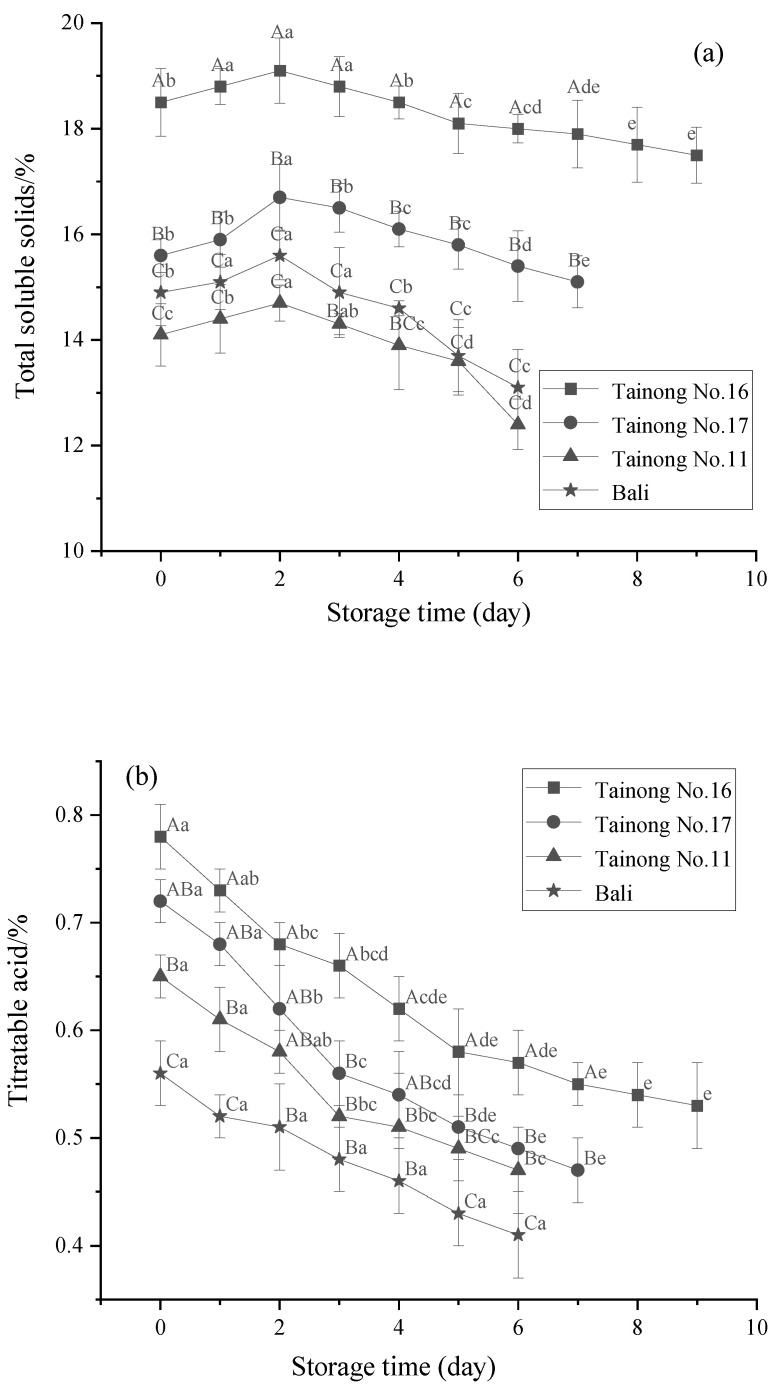

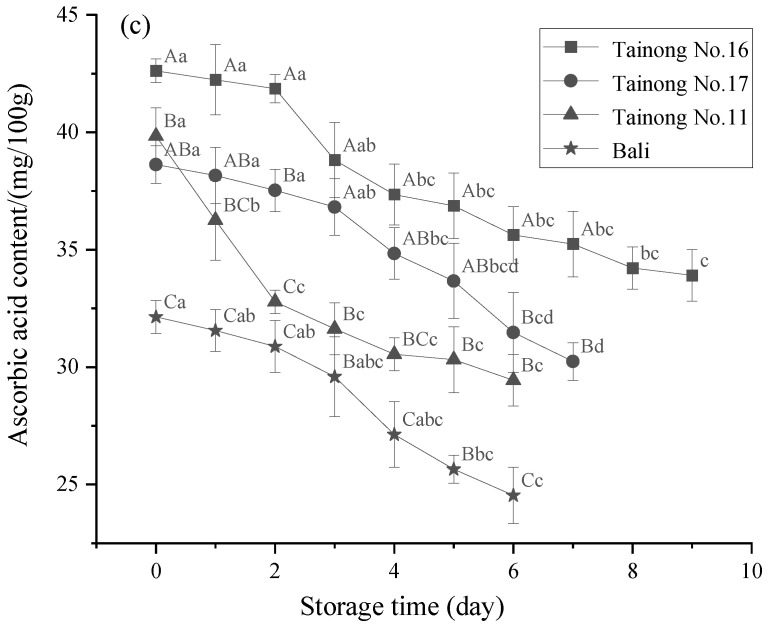
Changes in total soluble solids (**a**), titratable acid content (**b**), and ascorbic acid content (**c**) in freshly cut pineapple during storage. Different capital letters (A–C) indicate significant (*p* < 0.05) differences in total soluble solids, titratable acid content, and ascorbic acid content between varieties at the same storage time; different lowercase letters (a–e) indicate significant (*p* < 0.05) differences in total soluble solids, titratable acid content, and ascorbic acid content between the same varieties at different storage times.

The TSS content is commonly associated with eating quality and is always used as a quality criterion for selecting fruits suitable for fresh markets [43]. The TSS changes in the different freshly cut pineapple varieties during storage are shown in Figure 4a. The initial TSS content varied among the freshly cut pineapple samples, with the highest levels evident in *Tainong No.16* at 18.50%. After an initial increase in the TSS content of each variety during storage, this value decreased, followed by a significant decline after 2 d. The most significant change in the TSS value occurred in *Bali*, followed by *Tainong No.11*, *Tainong No.17*, and *Tainong No.16*, which declined by 16.03%, 15.65%, 9.58%, and 8.37%, respectively. In addition, the two-way interaction of variety and storage time (Table 2) had a significant effect (*p* < 0.05) on the TSS of the fresh-cut pineapple, and the main effect of variety on the TSS of the fresh-cut pineapple was the largest. The results indicated significant differences (*p* < 0.05) in the TSS levels of the four pineapple samples, which could be attributed to the role of the variety. TSS mainly include soluble sugars and organic substances, which constitute the primary substrate for respiration, while their content is closely related to the degree of fruit aging [44]. The overall TSS content initially increased, followed by a decrease, which could be due to the conversion of macromolecules, such as starch into soluble sugars, in the freshly cut pineapple samples during the early stage of storage, while significant sugar consumption was evident during the later stage as the respiration of the samples improved [45].

Figure 4b illustrates the changes in the TA content of the different pineapple varieties at various storage time points. The TA content displayed a similar variation pattern among all the samples, decreasing throughout the storage period. Significant differences (*p* < 0.05) were apparent between the initial TA content of the various pineapple samples (*Tainong No.16* > *Tainong No.17* > *Tainong No.11* > *Bali*). These differences could be ascribed to factors such as variety and cultivation conditions, which caused compositional variations in the fruit [46]. As shown in Table 2, the interaction between variety and storage time on TA was not significant (*p* > 0.05), and the main effect of storage time on the TA of fresh-cut pineapple was the largest. Previous studies have shown that the TA content is related to varietal differences. Fuentes-Pérez revealed significant differences between the TA levels of six yellow-fleshed peach varieties, with *Ruby Rich* displaying the highest content and *Royal Glory* the lowest, respectively [47]. Nogales-Delgado et al., indicated that different nectarine cultivars exhibited TA variation after being freshly cut, with *Venus* showing the highest acidity and *Nectaprima* and *Big Top* the lowest [48]. Moreover, TA loss increased with more extended storage periods, possibly indicating the utilization of organic acids as substrates for respiratory metabolism due to the accelerated respiration of injured tissues.

Pineapples are rich in AA, which plays a crucial role in many metabolic pathways, and directly affects the nutritional quality of freshly cut pineapple [49]. As shown in Figure 4c, the initial AA content values of the four pineapple samples varied substantially. *Tainong No.16* displayed the highest AA value, significantly exceeding the other varieties (*p* < 0.05). Consequently, these initial differences yielded final values of 33.91 mg/100 g, 29.24 mg/100 g, 29.44 mg/100 g, and 25.54 mg/100 g for *Tainong No.16*, *Tainong No.17*, *Tainong No.11*, and *Bali*, respectively. The AA content of each pineapple variety decreased with the extension of storage time, showing a dramatic decline from 0 to 4. *Tainong No.11* displayed the highest AA content loss, followed by *Bali*, *Tainong No.17*, and *Tainong No.16*, which decreased by 26.10%, 23.65%, 21.72%, and 20.44%, respectively. The AA content was significantly different among the four varieties (*p* < 0.05), in descending order: *Tainong No.16* > *Tainong No.17* > *Tainong No.11* > *Bali*. The main and interaction effects of variety and storage time on the AA of fresh-cut pineapple quality are shown in Table 3. It can be found that the interaction effect of variety and storage time on AA was not significant (*p* > 0.05) and the main effect of variety on the AA of fresh-cut pineapple was the largest.

The AA tended to decline during the storage of the freshly cut pineapples. This could be attributed to the fact that AA is a water-soluble vitamin. When the tissue structure of the pineapple is damaged and loses the protection of the dermis after the cutting process, the AA is oxidized and loses water [50]. The decline in the AA content was gradual during the later stages of storage, which was due to the low temperature slowing down the physiological metabolic activity of the pineapple and reducing the loss of AA. In addition, the variation in the AA content was because the samples were derived from different pineapple varieties. This was consistent with the results of Gil et al., who showed that the *Ataulfo* mango displayed an AA content of 80 mg/100 g, which was higher than that of other mango varieties [51]. Inglese et al., revealed that the different AA change rates varied between the freshly cut *Settembrina* and *Ottobrina* yellow-fleshed peach varieties with increasing storage time [52].

### 3.3. MDA Content

**Figure 5:**
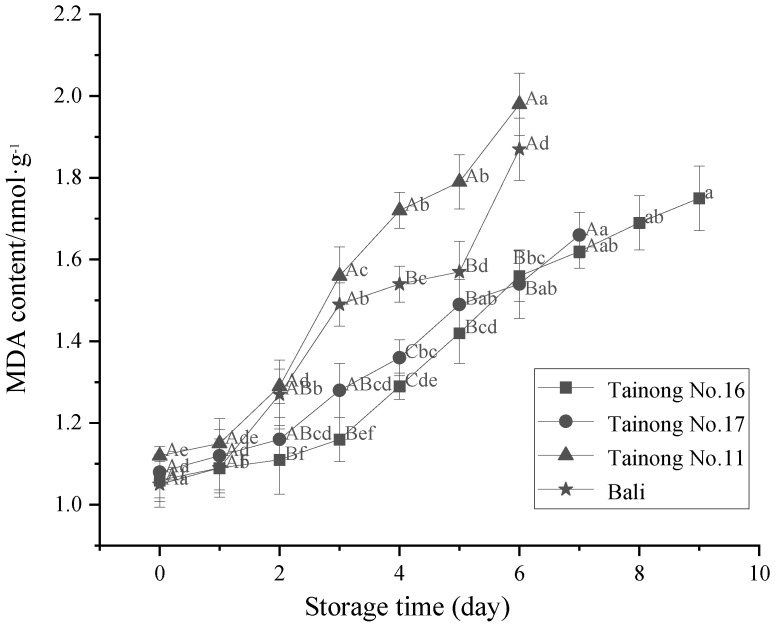
Changes in MDA content in freshly cut pineapple during storage. Different capital letters (A–C) indicate significant (*p* < 0.05) differences in hardness between varieties at the same storage time; different lowercase letters (a–f) indicate significant (*p* < 0.05) differences in hardness between the same varieties at different storage times.

As the final lipid peroxidation product, MDA is used as an index indicator to evaluate fruit senescence. Therefore, MDA, a secondary product of polyunsaturated fatty acid oxidation, denotes the degree of oxidative stress in plants [53]. The changes in the MDA content of the different freshly cut pineapple varieties during storage are shown in Figure 5. The results demonstrated that the MDA content of all varieties increased with extended storage time. At the beginning of storage, the MDA content of the pineapple samples displayed minimal differences. However, the MDA content varied significantly among the different varieties with extended storage time. The MDA content of *Tainong No.11* and *Bali* was considerably higher (*p* < 0.05) than the other two varieties at 0–6 d of storage. This could be attributed to the cut damage to the tissue cells of the freshly cut pineapple, substantially accelerating the rate of membrane lipid peroxidation, indicating that these two varieties were more susceptible to oxidative senescence [54]. The MDA content of *Tainong No.16* increased more rapidly than that of *Tainong No.17* up to 5 d of storage. This indicates that the physiological metabolism of *Tainong No.16* was more vigorous and suffered more damage from adversity until the later storage phase. Overall, *Tainong No.16* and *Tainong No.17* were more successful in maintaining normal cellular physiological functionality levels. These results indicate that storage time and varietal differences significantly affected membrane integrity, leading to different senescence levels in the freshly cut pineapples. The two-way interaction of variety and storage time (Table 2) had a significant effect on fresh-cut pineapple MDA (*p* < 0.05), and the main effect of storage time on fresh-cut pineapple MDA was the largest. Similar results were reported by Carvajal et al., who found that the MDA content in zucchini fruit increased with variety and storage time, while the rate of the increase varied [55].

### 3.4. PPO and POD Activity

**Figure 6:**
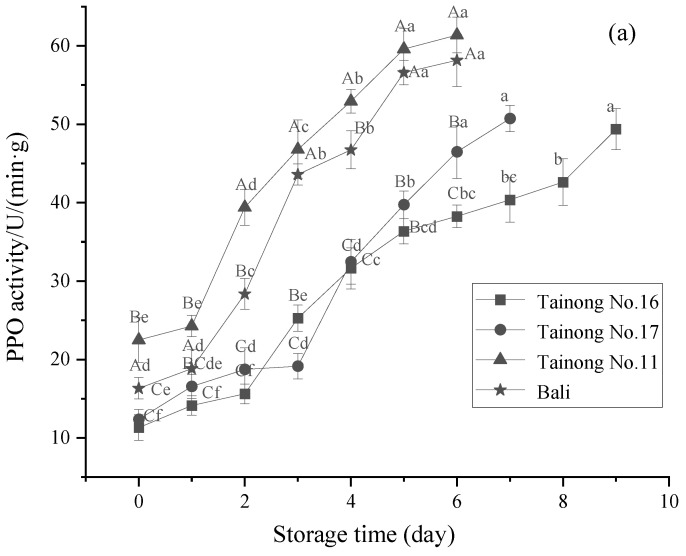

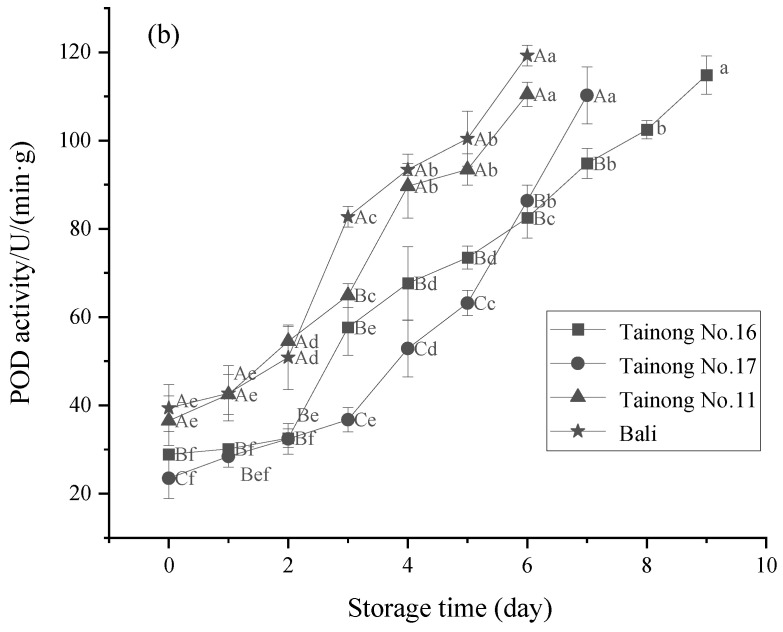
Changes of PPO activity (**a**) and POD activity (**b**) in freshly cut pineapple during storage. Different capital letters (A–C) indicate significant (*p* < 0.05) differences in PPO activity and POD activity between varieties at the same storage time; different lowercase letters (a–f) indicate significant (*p* < 0.05) differences in PPO activity and POD activity between the same varieties at different storage times.

PPO, a key enzyme for phenolic metabolism in fruits and vegetables, is widely distributed in plant cells and is significantly associated with browning [56]. Since PPO is responsible for post-cut browning in various fruits and vegetables, its changes in these pineapple cultivars were examined. The changes in the PPO activity of each pineapple variety during storage are shown in Figure 6a, revealing higher activity in each sample during storage. The maximum PPO-specific activity was observed in *Tainong No.11*, which was marginally higher than the other varieties in the 0–6 d sample, while it was highest in *Bali*. At the end of storage, the PPO enzyme activity of *Tainong No.16* was lower than the other three varieties (*p* < 0.05), and 17.67%, 37.66%, and 34.20% lower than that of *Tainong No.17*, *Tainong No.11*, and *Bali*, respectively. This indicated a variety-specific variation in the kinetics of the PPO activity during storage. Similarly, the two-way interaction of variety and storage time (Table 2) had a highly significant effect (*p* ≤ 0.001) on the PPO of fresh-cut pineapple, with storage time having the largest main effect on the PPO of fresh-cut pineapple. Furthermore, the PPO activity may vary due to gene sequences or epigenetics caused by differences in the cultivars [57]. Previous studies have indicated varietal differences in the PPO activity of other fruits and vegetables, including litchi, which may be due to variations in its levels of expression or bioactivity [58].

POD, a key enzyme for scavenging peroxides and promoting browning in fruits and vegetables, is closely related to physiological and biochemical metabolic processes [59]. As illustrated in Figure 6b, the POD activity of the freshly cut pineapple samples increased in all four varieties as the storage time was extended. *Bali* displayed the most significant change in POD enzyme activity throughout the storage period with an average daily increase of 13.31 U/min·g, which was significantly (*p* < 0.05) higher than the other varieties. The POD activity of *Tainong No.11* and *Bali* increased more rapidly after 1 d of storage, while that of *Tainong No.16* and *Tainong No.17* only became considerably higher after 2 d. The results revealed that the POD activity of freshly cut pineapple samples increased with storage time, representing the physiological response of the fruit to mechanical cutting and low-temperature refrigeration stress. The two-way interaction of variety and storage time (Table 2) had a highly significant effect (*p* ≤ 0.001) on the POD of the fresh-cut pineapple, with storage time having the largest main effect on the POD of the fresh-cut pineapple. POD activity is correlated with various deteriorative reactions, influencing the color, flavor, texture, and nutritional properties of processed fruits [60]. The overall POD activity increase indicated that the cutting damage destroyed the membrane structure. POD is considered an enzyme that eliminates radicals, which can promote browning [61]. Moreover, the variation in POD activity changes in the freshly cut pineapples could be attributed to varietal differences. Similar results were reported by Liu et al., who found that the POD activity of different types of fresh potatoes varied during refrigeration [62].

### 3.5. Microbiological Analysis

**Figure 7:**
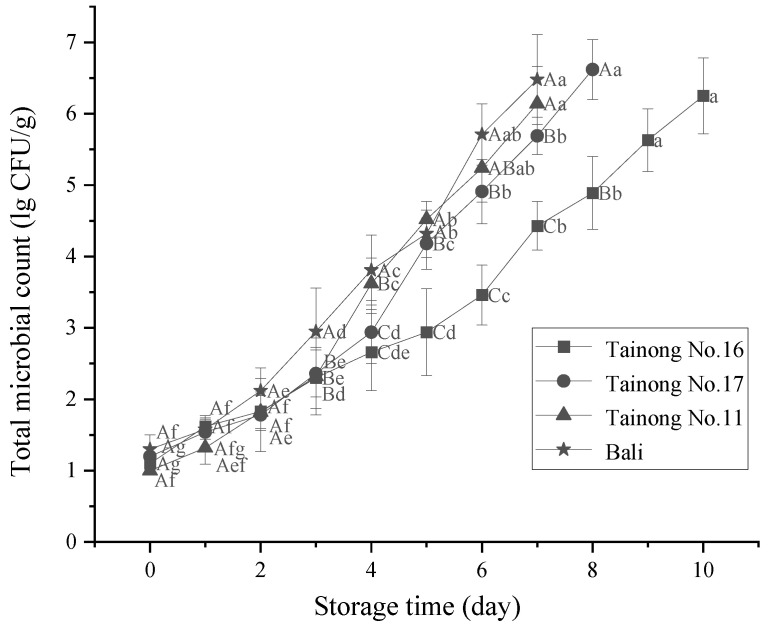
Changes in the total number of microorganisms in freshly cut pineapple during storage. Different capital letters (A–C) indicate significant (*p* < 0.05) differences in the total number of microorganisms between varieties at the same storage time; different lowercase letters (a–g) indicate significant (*p* < 0.05) differences in the total number of microorganisms between the same varieties at different storage times.

Microbial safety is crucial for preserving minimally processed fruits since aerobic bacterial counts reflect the freshness of the product and the sanitary conditions of the production facility [63]. The evolution of the aerobic microorganism counts of the freshly cut pineapple samples is presented in Figure 7. A total aerobic plate count showed that the total number of aerobic mesophilic microorganisms in all the samples was below the detection limit of 2.0 × 10^1^ CFU/g at 0 d. The low aerobic plate counts at the beginning of storage reflected the excellent quality of the raw materials. The aerobic plate counts gradually increased during the storage period. According to the limits established by the Food Safety and Standards Authority of India, 10^6^ CFU/g is considered the limit of acceptance of aerobic plate counts in cut or minimally processed fruits [64]. In the present study, the aerobic plate counts of *Tainong No.16* and *Tainong No.17* exceeded 10^6^ CFU/g after 10 d and 8 d of storage, respectively, while both *Tainong No.11* and *Bali* exceeded 10^6^ CFU/g after 7 d. Considering consumption safety and the current time limit for the cold chain sale of freshly cut pineapples, combined with the sensory assessment (Table 2), the total sensory score of 60 or less was selected as having no food value, so the storage time limits of 6 d, 5 d, 4 d, and 4 d were selected for *Tainong No.16*, *Tainong No.17*, *Tainong No.11*, and *Bali*, respectively. However, it is worth noting the lack of a previous sanitation step during the manufacturing process of the pineapple products, which could explain these discrepancies. Furthermore, the results showed (Table 2) that the two-way interaction of variety and storage time had a highly significant effect (*p* ≤ 0.001) on the total number of colonies of fresh-cut pineapple, with storage time having the largest main effect on the number of colonies of fresh-cut pineapple. Minimal processing allows for microbiota transfer through the surface to the flesh, increasing fruit spoilage [65]. Furthermore, freshly cut pineapples are an excellent source of nutrients for microbial growth since they are rich in water, sugars, and vitamins.

### 3.6. Evaluation of Sensory

As one of the most intuitive characteristics of fruits and vegetables, sensory characteristics are important reference indicators for consumer choice. The sensory analysis of the freshly cut pineapple samples during storage in terms of taste, smell, color, and texture is shown in Table 3. The results demonstrated that the taste, smell, color, and texture scores of these samples declined rapidly during storage. The initial sensory scores of all four pineapple varieties were different, with *Tainong No.16* displaying the highest total score of 97.66, presenting a better initial taste, smell, color, and texture than the other varieties. The overall acceptability of the freshly cut pineapple varieties was influenced by storage time. When stored for 0–2 d, the pineapples of each variety exhibited excellent freshness and sensory quality. After 3 d of storage, *Tainong No.11* and *Bali* exhibited textural softening and browning, while the sensory scores decreased significantly (*p* < 0.05). After 5 d of storage, *Tainong No.11* and *Bali* presented sticky surfaces, lost their consumption value, and displayed lower overall acceptability than *Tainong No.16* and *Tainong No.17*. The appearance of the pineapple samples was severely affected by microbial contamination at the end of storage due to the discoloration and wilting of the surfaces. The surfaces of *Tainong No.17* and *Tainong No.16* appeared sticky after 6 d and 7 d of storage, respectively. Therefore, *Tainong No.16* and *Tainong No.17* are more suitable for freshly cut storage. In addition, the results showed (Table 2) that the two-way interaction of variety and storage time had a significant effect (*p* < 0.05) on the sensory evaluation of fresh-cut pineapple, with storage time having the largest main effect on the sensory evaluation of fresh-cut pineapple. Therefore, the selection of pineapple varieties and the appropriate storage period are important factors affecting the quality of fresh-cut pineapples.

## 4. Conclusions

This study reveals significant differences in the initial sensory properties and nutritional content of freshly cut pineapples of different varieties. The quality assessment of these samples shows a decreasing trend throughout the storage period, with different physiological and biochemical changes in the four varieties. *Tainong No.16* presents a higher AA content, better weight maintenance, and more stable storage performance, making it more suitable for direct consumption. In comparison, the nutritional quality and resistance to the storage environment of *Tainong No.16* are superior to the other varieties after freshly cut processing, followed by *Tainong No.17*, *Tainong No.11*, and *Bali*. Furthermore, the varietal pineapple differences are closely related to the respective taste, nutritional quality, and storage characteristics of the freshly cut samples, highlighting the importance of selecting an appropriate variety for developing high-quality products. However, pineapple quality is influenced by storage conditions, soil characteristics, cultivation techniques, and regional characteristics. Therefore, further research is necessary to select pineapple varieties suitable for fresh consumption, processing, and cultivation.

## Figures and Tables

**Table 1:** Evaluation criteria for freshly cut pineapple sensory quality.

Quality Properties	Grade I	Grade II	Grade III
Taste (30 points)	Crunchy mouthfeel, moderately sweet and sour (20–30 points)	Taste brittle, sweet and sour discordant (10–19 points)	Soft, no obvious sweet and sour pineapple taste (0–9 points)
Smell (30 points)	Pineapple-specific aroma is strong and fresh smelling (20–30 points)	Lighter pineapple clear flavor, insufficient aroma (10–19 points)	Pineapple aroma is very light, with a peculiar smell (0–9 points)
Color (20 points)	Bright yellow, lustrous (14–20 points)	Light yellow, slightly shiny (7–13 points)	Yellowish brown or dark brown, no luster (0–6 points)
Texture (20 points)	Plenty of moisture and hard tissue (14–20 points)	Surface slightly dry and soft tissue (7–13 points)	Slightly putrid and soft (0–6 points)

**Table 2:** Main and interactive effects of variety and storage time on quality attributes of fresh-cut pineapple.

Variables		Cultivar	Storage Time	Cultivar·Storage Time
Weight loss	*p*-value	0.012	0.000	0.008
	Partial eta square	0.721	0.977	0.517
L* value	*p*-value	0.011	0.002	0.001
	Partial eta square	0.860	0.767	0.668
b value	*p*-value	0.024	0.006	0.003
	Partial eta square	0.881	0.792	0.691
firmness	*p*-value	0.000	0.001	0.000
	Partial eta square	0.935	0.858	0.957
TSS	*p*-value	0.031	0.001	0.009
	Partial eta square	0.923	0.912	0.400
TA	*p*-value	0.000	0.024	0.969
	Partial eta square	0.755	0.806	0.120
AA	*p*-value	0.000	0.001	0.790
	Partial eta square	0.781	0.726	0.175
MDA	*p*-value	0.006	0.000	0.003
	Partial eta square	0.622	0.912	0.429
PPO	*p*-value	0.005	0.001	0.000
	Partial eta square	0.910	0.972	0.660
POD	*p*-value	0.035	0.000	0.000
	Partial eta square	0.890	0.981	0.671
Microorganism	*p*-value	0.000	0.004	0.000
	Partial eta square	0.764	0.986	0.742
Evaluation of sensory	*p*-value	0.007	0.000	0.023
	Partial eta square	0.841	0.867	0.634

**Table 3:** Changes in sensory scores in freshly cut pineapple during storage.

Pineapple Cultivars	Parameter	Day 0	Day 1	Day 2	Day 3	Day 4	Day 5	Day 6	Day 7	Day 8	Day 9
*Tainong No.16*	Taste	29.42 ± 0.55 ^Aa^	29.11 ± 0.63 ^Aa^	28.61 ± 1.02 ^Aa^	28.24 ± 0.74 ^Aab^	27.21 ± 0.89 ^Ab^	23.26 ± 1.33 ^Ac^	21.88 ± 1.67 ^c^	—	—	—
	Smell	29.34 ± 0.55 ^Aa^	29.42 ± 0.49 ^Aa^	29.24 ± 0.75 ^Aa^	28.71 ± 0.41 ^Aab^	27.64 ± 0.48 ^Ab^	24.68 ± 1.02 ^Ac^	23.65 ± 0.48 ^Bd^	21.43 ± 0.48 ^Ad^	18.61 ± 1.02 ^d^	16.82 ± 0.75 ^e^
	Color	19.71 ± 0.41 ^Aa^	19.48 ± 0.49 ^Aa^	19.23 ± 0.41 ^Aa^	18.94 ± 0.20 ^Aa^	16.87 ± 0.40 ^Ab^	13.82 ± 1.12 ^Ac^	13.24 ± 0.74 ^Bc^	13.26 ± 0.63 ^Ac^	11.66 ± 1.37 ^d^	11.48 ± 0.63 ^d^
	Texture	19.21 ± 0.75 ^Aa^	19.22 ± 0.75 ^Aa^	19.01 ± 0.63 ^Aab^	18.81 ± 0.41 ^Aab^	17.92 ± 0.49 ^Ab^	14.46 ± 1.20 ^Ac^	13.78 ± 0.80 ^Bde^	13.24 ± 0.65 ^Ad^	12.31 ± 1.08 ^de^	11.85 ± 0.42 ^e^
	Score	97.66 ± 0.76 ^Aa^	97.23 ± 0.90 ^Aa^	96.01 ± 1.26 ^Aab^	94.62 ± 0.49 ^Ab^	89.35 ± 1.17 ^Ac^	76.14 ± 1.10 ^Bd^	71.67 ± 2.45 ^Be^	47.91 ± 0.63 ^Ae^	41.59 ± 1.99 ^f^	38.33 ± 1.20 ^g^
*Tainong No.17*	Taste	28.21 ± 0.74 ^Ba^	28.15 ± 0.63 ^Aa^	27.44 ± 0.49 ^Bab^	26.62 ± 0.82 ^Bbc^	25.89 ± 0.98 ^Ac^	20.26 ± 1.33 ^Bd^	—	—	—	—
	Smell	28.62 ± 0.48 ^Aa^	28.63 ± 1.03 ^Aa^	27.56 ± 0.45 ^Ba^	27.45 ± 0.48 ^Ba^	25.81 ± 0.98 ^Bb^	24.83 ± 0.75 ^Abc^	19.26 ± 0.75 ^Ac^	16.47 ± 1.50 ^Ad^	—	—
	Color	19.23 ± 0.42 ^ABab^	19.65 ± 0.36 ^Aa^	18.67 ± 0.48 ^ABab^	18.35 ± 1.10 ^Ab^	16.36 ± 1.41 ^Ac^	15.73 ± 1.09 ^Bcde^	14.42 ± 1.02 ^Ade^	13.62 ± 1.02 ^Bf^	—	—
	Texture	19.64 ± 0.48 ^Aa^	19.21 ± 0.47 ^Aa^	18.89 ± 0.74 ^Aa^	17.26 ± 0.75 ^Bb^	15.82 ± 0.75 ^Bc^	16.37 ± 1.25 ^Abc^	15.86 ± 0.75 ^Ac^	13.43 ± 1.34 ^Bd^	—	—
	Score	95.66 ± 1.01 ^ABa^	95.43 ± 1.49 ^Aa^	92.35 ± 1.07 ^Bb^	89.28 ± 1.72 ^Bc^	83.45 ± 2.80 ^Bd^	75.67 ± 2.61 ^Ae^	49.59 ± 0.81 ^Af^	43.77 ± 2.25 ^Bg^	—	—
*Tainong No.11*	Taste	27.53 ± 0.63 ^BCa^	26.66 ± 1.02 ^Ba^	26.24 ± 0.74 ^Ca^	23.25 ± 1.33 ^Cb^	19.82 ± 0.67 ^Bc^	—	—	—	—	—
	Smell	29.32 ± 1.09 ^Aa^	28.62 ± 1.34 ^Aa^	28.42 ± 0.83 ^ABa^	26.53 ± 0.89 ^Cb^	23.86 ± 0.74 ^Cc^	19.20 ± 1.38 ^Bd^	16.63 ± 0.86 ^Cd^	—	—	—
	Color	18.84 ± 0.74 ^Ba^	17.44 ± 0.86 ^Bb^	17.45 ± 1.03 ^BCb^	16.22 ± 1.17 ^Bb^	13.68 ± 1.65 ^Bc^	12.52 ± 0.63 ^Cd^	11.28 ± 0.74 ^Cd^	—	—	—
	Texture	18.66 ± 0.48 ^Aa^	17.96 ± 0.9 c2 ^Bab^	18.48 ± 0.82 ^ABa^	16.65 ± 1.02 ^Bb^	12.42 ± 1.62 ^Bc^	11.18 ± 0.66 ^Bcd^	10.43 ± 1.06 ^Cd^	—	—	—
	Score	93.49 ± 1.62 ^Ba^	90.59 ± 1.73 ^Ba^	90.40 ± 1.36 ^Ba^	82.12 ± 2.28 ^Cb^	69.64 ± 2.42 ^Cc^	42.52 ± 2.49 ^Cd^	37.68 ± 2.24 ^Cd^	—	—	—
*Bali*	Taste	26.83 ± 1.17 ^Ca^	26.51 ± 0.63 ^Ba^	26.52 ± 0.63 ^Ca^	23.61 ± 1.28 ^Cb^	18.62 ± 1.85 ^Bc^	—	—	—	—	—
	Smell	26.86 ± 1.33 ^Ba^	26.44 ± 1.01 ^Ba^	26.62 ± 0.89 ^Ca^	25.45 ± 1.23 ^Ca^	22.54 ± 0.89 ^Db^	18.81 ± 0.75 ^Bc^	15.56 ± 0.89 ^Cc^	—	—	—
	Color	18.58 ± 1.45 ^Ba^	18.29 ± 0.75 ^Ba^	17.88 ± 0.76 ^Ca^	15.97 ± 0.66 ^Bb^	14.47 ± 1.02 ^Bc^	12.63 ± 1.06 ^BCd^	11.22 ± 0.75 ^Ce^	—	—	—
	Texture	18.82 ± 0.75 ^Aa^	18.64 ± 0.78 ^Ba^	17.42 ± 1.02 ^Bab^	16.83 ± 1.09 ^Bbc^	15.21 ± 0.25 ^Cc^	12.35 ± 1.66 ^Bd^	12.11 ± 0.66 ^Bd^	—	—	—
	Score	90.94 ± 2.42 ^Ca^	89.28 ± 0.74 ^Bab^	87.29 ± 2.71 ^Bb^	80.99 ± 2.76 ^Cc^	70.71 ± 3.68 ^Cd^	42.83 ± 1.72 ^Ce^	38.39 ± 1.40 ^Ce^	—	—	—

Different capital letters (A–D) indicate that taste, smell, color, texture, and score for different varieties were different significantly (*p* < 0.05) at the same storage time; different lower case letters (a–d) indicated that taste, smell, color, texture, and score for different storage times were different significantly (*p* < 0.05) at the same varieties. —: Not tested.

## Data Availability

Not applicable.
